# Computed Tomography Assessment of Retained Testes in Dogs †

**DOI:** 10.3390/ani15101439

**Published:** 2025-05-16

**Authors:** Stefano Spada, Daniela De Felice, Alfonso Calabria, Francesca Carletti, Luiz Paulo Nogueira Aires, Massimo Vignoli, Martina Rosto, Marco Russo

**Affiliations:** 1 Clinic of Reproductive Medicine, Vetsuisse Faculty of Zurich, University of Zurich, 8057 Zurich, Switzerland; 2 Department of Veterinary Medicine and Animal Production, University of Naples, Federico II, 80137 Naples, Italy; daniela.defelice2@unina.it (D.D.F.); alfonso.calabria@unina.it (A.C.); marco.russo@unina.it (M.R.); 3 Department of Veterinary Medicine, University of Teramo, 64100 Teramo, Italy; francesca.carletti@studenti.unite.it (F.C.); mrosto@unite.it (M.R.); 4 School of Agricultural and Veterinarian Sciences, São Paulo State University “Júlio de Mesquita Filho” (FCAV/UNESP), Jaboticabal 14884-900, São Paulo, Brazil; luiz.aires@unesp.br

**Keywords:** cryptorchidism, dog, computed tomography, testicles, advanced imaging

## Abstract

Cryptorchidism consists in the failure of one or both testicles to fully descend into the scrotum. The position of the retained testes can be abdominal or inguinal and may occur unilaterally or bilaterally. This retrospective, multi-center study aimed to describe the computed tomography (CT) features of retained testes in dogs. Nineteen CT scans of dogs, with either unilateral or bilateral cryptorchidism, were analyzed with both pre- and post-contrast imaging. The location, size, shape, margins, homogeneous parenchyma, and density calculated were examined with the Hounsfield unit. Statistical analyses were performed to assess the differences between the scrotal (ST) and undescended testes (UT) and to detect any correlation between the features, ages, and size of the dog. CT identified the retained testes and provided enhanced three-dimensional visualization compared to traditional ultrasound. This study revealed that the UT were significantly smaller than ST, with UT measuring around 70% of ST size. Additionally, the UT exhibited increased density in both pre- and post-contrast scans, potentially due to the reduced or absent spermatogenesis or to histological changes occurring in the parenchyma. While CT showed clear advantages, such as three-dimensional spatial resolution and deeper tissue penetration, limitations such as general anesthesia and radiation exposure should also be considered. However, the present study showed that CT could serve as a valuable second step tool in cases where ultrasound fails, particularly in challenging anatomical situations.

## Simple Summary

Cryptorchidism is a condition in which one or both testicles fail to move into their normal position in the scrotum, which can affect dogs on one or both sides. This study analyzed computed tomography scans of 19 dogs with cryptorchidism. The results showed that undescended testicles were smaller than normal ones, measuring about 70% of their size, and they had higher density, possibly due to reduced sperm production or structural changes. CT provided clearer, more detailed images than ultrasound, making it a useful tool when ultrasound is inconclusive, especially in complex cases. However, CT requires anesthesia and exposes dogs to radiation, which are important considerations. Despite these drawbacks, the findings suggest that CT can be a valuable second-line imaging method to improve diagnosis and treatment planning for affected dogs. This could help veterinarians make better decisions, ensuring timely and appropriate care while minimizing complications related to undiagnosed retained testicles.

## 1. Introduction

Cryptorchidism consists in the uncomplete descent of one or both testicles inside the scrotal sac and has an incidence of 6.8% in dogs [1,2]. Retained testes may be abdominal or inguinal, with the right unilateral inguinal region being the most frequent in dogs, similar to occurrences in humans [3,4]. In the case of abdominal testes, they may be localized adjacent to the kidneys in the retroperitoneum or anywhere along the path of testicular descent into the dependent hemiscrotum [5,6]. Recent studies have demonstrated the hereditary basis of the present condition [7], whose transmission probably involves more than one gene [8,9]. Cryptorchidism is of clinical concern in routine vet practice since several studies have shown an increased potential of retained testicular tissue developing neoplastic changes [1,2,9,10], with interstitial cell tumors, seminoma, and Sertoli cell tumors being the most represented neoplasia [11], and testicular torsion being another likely eventuality [12,13].

The diagnosis of cryptorchidism and the localization of retained testes can be challenging. It is usually based on the patient’s history, especially regarding the neuter status and palpation of the scrotum or inguinal region [14]. Ultrasound is a non-invasive and widely available sensitive technique that is used to detect retained testes in horses [15,16,17], dogs, and cats [6]. Felumlee and colleagues (2012) found a 100% and 96.6% of positive predictive value of ultrasonography to locate inguinal and abdominal testes, respectively. However, the ability to identify a non-palpable testicle may be affected by several factors, such as the experience of the operator, the dog and testicle’s size, and the normal appearance of the retained testes [6]. In children, it has been observed that the sensitivity and specificity of ultrasound in correctly identifying a non-palpable testis is 45% and 78%, respectively [18]. The presence of a hyperechoic mediastinum testes and capsule; echogenic parenchyma; the presence of the head, body, and tail of the epididymis; and the identification of pampiniform plexus by color Doppler are key features to consider of the imaged structure as a testicle [6,19,20]. However, morphological alterations frequently occur in retained testes [21], and ultrasonographic features may not be present or may appear different [6]. In particular, abdominal lymph nodes may be characterized by similar ultrasonographic appearance, especially in young dogs, such as increased size, ellipsoid shape, hyperechoic center, and hypoechoic parenchyma, with jejunal ones being bigger in younger than adult dogs [22]. Lack of experience or the different ultrasonographic appearance of a retained testicle may significantly affect the ability to recognize it. Computed tomography (CT) offers a three-dimensional overview of the abdomen and pelvis, enabling an improved visualization of the anatomical structures. Unfortunately, only one study has investigated the potential use of CT for assessing retained testes in veterinary medicine. Goda and colleagues (2022) evaluated the use of CT in the assessment and location of retained testes in bulls, showing promising results for an appropriate surgical planning [23]. However, data concerning the usefulness of CT in the identification and location of undescended testes in dogs are still scant.

The purpose of the present study was to explore the potential of CT in the assessment of retained testes in dogs. We hypothesize that CT may provide new and valuable insights into the differences between retained and scrotal testicles, as well as its potential to accurately locate undescended testicles.

## 2. Materials and Methods

### 2.1. Study Design and Case Selection

For the present multicenter retrospective anatomical study, CT scans of cryptorchid dogs were retrospectively enrolled for the present study, including cases from May 2014 to March 2024 presented for whole body, abdominal, and/or pelvic diagnostic imaging in two different private clinics: “Veterinary Clinic PetCare” (Bologna (BO), Italy) and “Calabria Veterinary Center” (Marigliano (NA), Naples, Italy). The medical records were searched to identify the CT studies of dogs affected by cryptorchidism. Search terms included “cryptorchidism”, “retained testes”, “undescended testes”, and “cryptorchid dog”. Criteria for inclusion in the CT studies were as follows: both testicles had to be present within the study, CT examination should have included a pre- and post-contrast study, and no neoplastic conditions affecting the testicles should have been detected. For every dog, the breed, age, and weight was recorded. For the present study, no ethical approval was required as it was a retrospective analysis of clinical procedures already performed in the past without any additional sampling or unnecessary interventions. Furthermore, all data were anonymized, with the exception of the age and weight of the dogs.

### 2.2. CT Scan Examination

For the CT studies, all of the dogs had been anesthetized and positioned in sternal recumbency. The anesthetic protocol was consistent across all specimens, with slight differences due to the clinical conditions of the patients. Two 16-slice multidetector CTs (BrightSpeed GE and Optima 540 GE, Milwaukee, WI, USA) were consistently employed for all scans, with the gantry remaining untilted and a slice thickness ranging between 1.25–2.5 mm, pitch 1.3, and voltage 120 kVp. The field of view was adjusted to a size that included the whole abdomen. All settings were the same for the pre- and post-contrast scans. The contrast study was performed 40 s following the injection of a non-ionic iodinated contrast agent (Optiray, Guerbet, Roissy, France) in the cephalic vein using a contrast injector (Medrad Vistron CT^®^ injection system, Indianola, PA, USA) at a dose of 600 mg/kg. Soft tissue settings (WW 300–350, WL 35–40) were utilized for review.

### 2.3. CT Scan Interpretation

All CT studies were evaluated under the supervision of a board-certified veterinary radiology specialist (M.V.) for the identification of the testicular tissue and surrounding structure. Images were initially reviewed on a PACS viewer (picture archiving and communications system) before being uploaded to Horos DICOM Viewer software for further processing and analysis (Horos © 2020, The Horos Project & OsiriX Team, version 3.3.6). For each dog, CT data were available as transverse image stacks. Sagittal and dorsal images were reconstructed using Horos software (v.3.3.6). Display settings were kept constant, with a window width of 300 Hounsfield units (HU) and a window level of 30 HU. Both testicles were identified and evaluated in terms of location, size, shape, margins, and density, which were measured in HU and parenchymal homogeneity. Based on the CT examination, the dogs were divided into two groups, being bilateral or unilateral cryptorchid dogs. To indicate the location of the abdominal testicles, a sagittal view was used and the corresponding vertebra on the same orthogonal plan of the undescended testes (UT) was identified. Using multiplanar reconstructions (MPR), the testicular length (cranio-caudal), height (dorso-ventral), and width (latero-lateral) were measured in cm to obtain an overall size of the testicles. Based on the results, a ratio was then established for every single measurement in order to determine the difference between the scrotal (ST) and UT in each patient. The shape, margins, and parenchymal homogeneity was described subjectively. The testicular HU was measured by placing a circular 2.5 cm^2^ ROI (region of interest) on both testicular parenchyma and pampiniform plexus in the pre- and post-contrast studies. The increase in terms of HU between the pre- and post-contrast studies was calculated both in parenchyma and plexus as the difference between the HU post- and HU pre-contrast values. The prostate gland was also evaluated subjectively as part of a complete evaluation of the male reproductive tract.

### 2.4. Statistical Analysis

Data were initially recorded using a computerized spreadsheet (Microsoft Excel 2021) and then imported into Statistical Package for Social Sciences (SPSS IBM Statistics version 27.0, IBM Corporation, Armonk, NY, USA) for statistical analysis. The normality of the data distribution was tested using the Shapiro–Wilk test. The variables were not normally distributed, and the results were reported as the median and interquartile range.

A Mann–Whitney test was performed to compare the median weight between the dogs with left vs. right cryptorchidism and with abdominal vs. inguinal cryptorchidism. A Spearman test was then used to assess a potential correlation between the weight and age of the dogs, as well as to assess the following:

- The HU of the UT and ST parenchyma and plexus both pre- and post-contrast administration;
- The linear measurements of the UT and ST.

The correlation was considered weak, moderate, strong, or perfect when the value of the correlation coefficient was 0.1−0.3, 0.4−0.6, 0.7−0.9, or 1, respectively. For all statistical tests, the level of significance was set at *p* < 0.05.

The Mann–Whitney test was then used to assess the following:

- The differences in terms of the size and HU between the UT and ST within the same specimen in unilateral cryptorchid dogs.
- The differences between the left and right testes in bilateral cryptorchid dogs.
- The difference in the increase in the HU from the pre- to post-contrast studies of the parenchyma and plexus between UT and ST.

A Wilcoxon signed rank test was then used to assess the differences in terms of the HU of the testes between pre- and post-contrast administration studies.

## 3. Results

### 3.1. Animals

Medical records of 29 cases were evaluated for this study, but only 19 cases met the inclusion criteria. The other 10 cases were excluded due to the absence of a contrast study, the absence of both testicles, or the presence of a neoplastic condition affecting the testicles. The patients underwent a CT scan examination for several clinical reasons not affecting the genitourinary tract. Moreover, the anesthetic protocol for all dogs consisted in using an alfa-2-agonist, such as medetomidine or dexmedetomidine, and opioids, such as methadone for premedication, propofol for anesthetic induction, and sevoflurane for maintenance of the anesthesia after intubation.

Of the 19 dogs, 15 were classified as unilateral cryptorchid, with 8 having abdominal cryptorchidism and 7 having inguinal cryptorchidism. In particular, the retained testes were more frequently observed on the right side (9 right and 6 left side). The remaining four dogs were bilateral cryptorchid, comprising two with bilateral abdominal cryptorchidism, one with bilateral inguinal cryptorchidism, and one with one testicle located in the abdomen and the other in the inguinal canal. The dogs recruited for the present study were of different breeds: German Shepherd (3), Golden Retriever (2), Pinscher (2), Dachshund (2), French Bulldog (2), Jack Russell (1), Chihuahua (1), Maremma Sheepdog (1), Pitbull (1), Poodle (1), Cocker (1), a Border Collie (1) and a mix breed (1). The median weight and age of the recruited specimen were 17 kg (IQR = 8–34) and 36 months (IQR = 18–84), respectively. Weight did not show any influence on the localization of the testes in terms of abdominal vs. inguinal (*p* = 0.35) or left vs. right (*p* = 0.72) cryptorchidism.

### 3.2. CT Testicles

**Figure 1:**
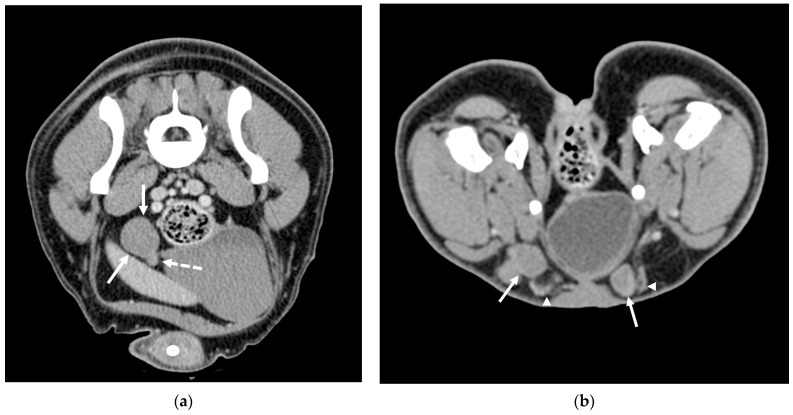
CT scans of two cases of cryptorchidism: (**a**) Post-contrast image showing an abdominally retained testicle (solid arrows), which appears homogeneously enhanced. The dashed arrow indicates the epididymis and pampiniform plexus, which show higher density compared to the testicular parenchyma. (**b**) Post-contrast scan of two inguinal testicles (solid arrows) with a visible pampiniform plexus (arrowheads).

CT successfully identified all testicles, both abdominal and inguinal. The positions of the abdominal testicles varied significantly in relation to the median sagittal line; they were observed either deeply and centrally near the aorta and vena cava or superficially underneath the fascia (Figure 1). On transverse scans, their locations ranged between the sixth lumbar vertebra and the first sacral vertebra.

**Figure 2:**
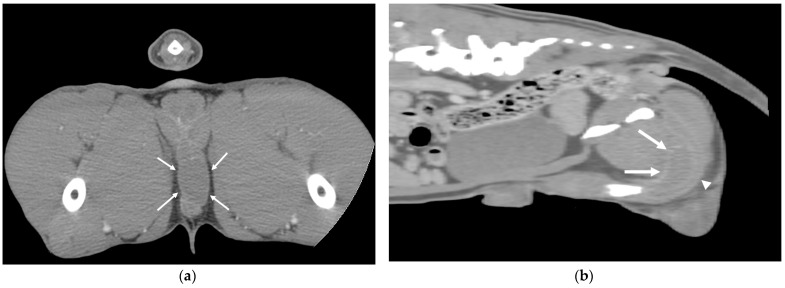
Post-contrast scan of the axial (**a**) and sagittal view (**b**) of a retained perineal testicle (solid arrows). The testicle was located cranio-dorsal to the urethra and corpus spongiosum of the penis (arrowhead).

Interestingly, one dog excluded from the present study due to having only one testicle (having previously undergone unilateral orchiectomy of the scrotal testis) exhibited an unusual and distinctive testicular position. In this case, the testicle was located in a perineal position, cranial to the penile corpus spongiosum and caudal to the pelvic diaphragm (Figure 2).

The UT were statistically smaller than ST in all cases and in all three linear measurements (H, *p* = 0.04; L, *p* = 0.004; W, *p* = 0.001) than the contralateral scrotal testicle of about 30%, which was assessed using ratios. Parameters concerning the linear size measurements of both scrotal and cryptorchid testes are reported in Table 1.

The shape of both UT and ST was ellipsoid for all dogs, with a mild tendency to be more ovoidal in case of UT, as can be seen by the lower ratio of the testicular length. The epididymis was similar in appearance in both UT and ST. The ductus deferens, the pampiniform plexus, and the testicular artery and vein could be identified both with and without contrast administration in both UT and ST. Parenchymal differences were detected, with UT having a higher HU in both pre- (*p* = 0.026) and post-contrast (*p* = 0.044) studies when compared to the ST. No differences were detected when looking at the plexus density between the two groups. In both groups, both the plexus and parenchyma HU increased from the pre- to post-contrast studies. No difference was detected when looking at the increase in the HU from the pre- to post-contrast studies of both the parenchyma and plexus between UT and ST. The testicular HU values of the population are reported in Table 2.

Spearman tests revealed a moderate negative correlation between the age and HU measured in the UT parenchyma in pre-contrast studies (*r* = −0.63; *p* = 0.01), as well as a positive moderate correlation between the age and HU measured in the pampiniform plexus of the UT in post-contrast studies (*r* = 0.6; *p* = 0.02).

Moreover, Spearman tests showed a positive correlation between the weight and parenchymal HU of ST pre-contrast (*r* = 0.82; *p* = 0.001); the parenchymal HU of ST post-contrast (*r* = 0.62; *p* = 0.014); and the H, L, and W of both the UT (*r* = 0.89, *p* = 0.001; *r* = 0.6, *p* = 0.02; *r* = 0.51, *p* = 0.05) and ST (*r* = 0.83, *p* = 0.001; *r* = 0.84, *p* = 0.001; *r* = 0.7, *p* = 0.005).

The prostate gland appeared in all dogs as a butterfly-shaped gland in transverse view and ovoidal in longitudinal view. The lobes were symmetric in most of the cases, except in prostates affected by benign prostatic hyperplasia.

Parenchymal abnormalities were detected in eight dogs out of nineteen and were as follows: six with inhomogeneous parenchyma, one with prostatic abscesses, and two cases with paraprostatic cysts. Parenchymal abnormalities were better evaluated after contrast administration, which enabled the differentiation of the parenchyma from cystic structures.

## 4. Discussion

The present study highlights the promising potential of CT imaging in locating and evaluating retained testes in dogs. Specifically, this study demonstrated that the three-dimensional visualization of the abdomen and inguinal regions provided by CT enables the identification of retained testicles and may improve their localization. Moreover, the present study showed new insights into the features of UT and ST in dogs, introducing a specific ratio for testicular size and reporting difference in terms of the testicular tissue density in UT. Although ultrasound is the standard diagnostic tool for locating retained testes, in the experience of the author, it can be particularly challenging, especially in very large or overweight dogs. In addition, previous studies have reported a high success rate for ultrasound in detecting retained testes, but these findings may not accurately reflect real clinical practice as they did not account for operator experience or the characteristics of the specimens examined [6]. Moreover, locating retained testes may represent a challenge, even for highly experienced ultrasonographers, due to their variable positioning and changes in size and appearance, as was observed during the present study.

In this study, the position of the abdominal testes varied significantly among the specimens, ranging from superficial locations to positions near the sagittal median line. Due to the retrospective nature of this study, a direct comparison between the CT imaging and the widely used ultrasonography technique was not possible. However, in some cases, the testicles were identified medially to the colon, a position that can pose significant challenge using ultrasound. However, even though we managed to determine the potential location of a retained testicles using the orthogonal plane, it should be emphasized that the position may vary substantially across the population. A notable case was the perineal position of a retained testicle in one dog, which was excluded from the present study. In humans, ectopic perineal testicular positioning is a rare abnormality of testicular descent, accounting for approximately 1% of retained testicle cases [24]. Ectopic testicles deviate from the normal descent pathway, with possible locations including the superficial inguinal pouch, perineum, penis, lateral scrotum, pubic region, thigh, or contralateral scrotum [25]. To our knowledge, this is the first reported case of an ectopic perineal testicle in a dog, underscoring the challenges associated with identifying retained testes. In particular, the unusual location may significantly impair the ability of ultrasonography to detect the testicle, emphasizing the potential advantage of CT in such complex diagnostic cases.

In the present study, we observed a significant difference in terms of the size between UT and ST, confirming previous results from other studies [2,21,26]. In particular, we found that the UT was approximately 70% of the size of the contralateral ST, although shape and margins remained consistent between the two groups. This size mismatch may be due to the reduced or complete absence of spermatogenesis in the seminiferous tubules and abnormalities of both Sertoli and Leydig cells within retained testes. In particular, several studies have identified peculiar histological features in UT, such as seminiferous tubules without mature germ cells called Sertoli cells only tubules (SCO) and Sertoli cells hyperplasia [21,26,27]. However, besides the different size, both UT and ST showed positive correlation with the dog’s weight. The observed correlation between body weight and testicular size is clinically relevant as it suggests that retained testicles in larger dogs may be more readily detectable on imaging due to their increased dimensions. Conversely, another interesting finding from the present study was the negative correlation found between the age and the HU values of UT in pre-contrast parenchyma. Several studies have reported that timing exposure to increased temperature is crucial for neoplastic development as, in children, the risk for testicular malignancy reduces when orchidopexy is performed before puberty, although it does persist despite surgical treatment [28]. We may speculate that parenchymal changes can occur over time as degenerative processes of senescence, or due to potential neoplastic development, could explain the negative trends of the UT HU in older dogs. These findings raise questions about whether tissue density alterations could impact diagnostic accuracy, particularly in older cryptorchid dogs. Further investigation is needed to determine whether such changes affect surgical decision making or the likelihood of testicular pathology.

The peculiar histological features of UT may also explain the increased density detected in the latter compared to ST in both pre- and post-contrast studies. The absence of semen may alter the density of the tissue, especially due to the reduced quantity of water. Indeed, Moxon and colleagues reported the potential impact of sperm production on the ultrasonographic echogenicity and heterogeneity of testicular tissue [29]. Moreover, the water imbalance in UT has been also suggested in previous studies that found different expressions of Aquaporins in normal ST and UT [30,31].

Even though information concerning the histological characteristics of UT parenchyma are available, to our knowledge, there are no studies focusing on the vascular differences between ST and UT. In our study, the UT also showed an increased post-contrast density when compared to ST, even though we did not find any difference in terms of the HU increase between UT and ST, suggesting that the contrast agent enhanced the tissue in a similar way in both groups. Nevertheless, it has been reported that an atypical position may determine displacement of the pampiniform plexus [20], which may lead to differences in terms of vascular dynamics, flow resistivity, and pressure within the testicles. This could support our findings concerning the increased pampiniform plexus HU in post-contrast studies of older dogs.

The results from the present study showed that CT offers distinct advantages over ultrasound, including superior spatial resolution, deeper tissue penetration, and the ability to generate three-dimensional reconstructions. While ultrasound remains a commonly used modality due to its accessibility and lack of ionizing radiation, it is highly operator-dependent and may be limited by patient factors, such as obesity or excessive bowel gas. Compared to magnetic resonance imaging (MRI), CT provides faster image acquisition, making it a more practical option in clinical settings, although MRI may offer superior soft tissue contrast in certain scenarios. However, we could not compare the two techniques as this was not within the scope of the present study. Further studies concerning the ability of both ultrasound and CT techniques to locate and evaluate retained testicles should be conducted. Nonetheless, it should be emphasized that CT is not without limitations. The need for sedation or general anesthesia, exposure to ionizing radiation, and cost considerations may limit its widespread use as a first-line diagnostic tool. In actuality, few studies are available in human medicine concerning the use of CT in the identification of undescended testicles [32,33] due to the risk of secondary malignancies conferred by ionizing radiation, which is especially pronounced in the pediatric population [34]. Unlike CT, MRI does not involve ionizing radiation and, therefore, represents an optimal diagnostic technique for pediatric patients [18]. Despite these challenges, in cases where ultrasound fails to provide definitive localization, such as in overweight patients or when testicles are intra-abdominal and deeply embedded, CT can serve as a valuable alternative.

Furthermore, the precise localization of retained testicles using CT has significant implications for surgical planning. Identifying the exact position of an undescended testicle before surgery can reduce exploration time, minimize surgical trauma, and improve overall outcomes. Testicular tumors can grow considerably large, making it extremely challenging to determine their size, position, relationship with adjacent structures, and potential metastatic spread [12,13]. Another advantage of CT is its ability to accurately assess the size of retained testes, which can be difficult with ultrasound, particularly when visualization is suboptimal due to their location. CT may provide more precise measurements, which can influence surgical decision making, especially in cases requiring laparoscopic approaches, where length for celiotomy incision is crucial [35]. Despite the promising findings, limitations concerning the present study exist, including a relatively small sample size, the lack of histological examination of the testicles, and its retrospective design. A larger, prospective study directly comparing CT and ultrasound in the evaluation of cryptorchidism would help validate these findings. Moreover, no histological findings are available to confirm CT results and to rule out any neoplastic changes occurring in the testicles. Future research could also explore optimized imaging protocols to minimize radiation exposure while maintaining diagnostic accuracy.

## 5. Conclusions

In conclusion, the present study suggests CT as a valuable and highly accurate method for localizing retained testicles in dogs, with potential benefits for surgical planning and the diagnosis of testicular abnormalities. While ultrasound remains the most commonly used modality, CT may be particularly beneficial in cases where ultrasound is inconclusive or when additional anatomical detail is required. Further research is warranted to refine its clinical application and to evaluate its cost–benefit ratio in veterinary practice.

## Figures and Tables

**Table 1:** The median and interquartile range of the size of the testes in both groups and the ratio of the single measurements between the two groups.

	Scrotal Testes (ST Group)	Undescended Testes (UT Group)	Ratio
Testicular Height	2.41 cm (IQR = 1.66–3.64)	1.67 cm (IQR = 0.99–2.25)	0.7 (IQR = 0.6–0.83)
Testicular Length	3.46 cm (IQR = 2.8–5.4)	2.27 cm (IQR = 1.41–3.45)	0.64 (IQR= 0.52–0.78)
Testicular Width	2.07 cm (IQR = 1.8–2.22)	1.43 cm (IQR = 1.14–1.61)	0.71 (IQR= 0.59–0.88)

**Table 2:** The median and interquartile ranges of the HU measured in the parenchyma and plexus pre- and post-contrast administration in both groups.

	Scrotal Testes (ST Group)	Undescended Testes (UT Group)
HU Parenchyma Pre-contrast	29.7 (IQR = 25.79–32.24)	36.75 (IQR = 31.13–45.09)
HU Parenchyma Post-contrast	42.14 (IQR = 36.1–48.83)	48.73 (IQR = 43.7–61.87)
HU Pampiniform Plexus Pre-contrast	37.96 (IQR = 33.89–44.79)	40.90 (IQR = 35.05–46.27)
HU Pampiniform Plexus Post-contrast	61.39 (IQR = 53.95–80.8)	69.11 (IQR = 61.34–81.99)

## Data Availability

The raw data supporting the conclusions of this article will be made available by the authors on request.

## Abbreviations

The following abbreviations are used in this manuscript:

UT	Undescended testes
ST	Scrotal testes
CT	Computed tomography
HU	Hounsfield unit
H	Height
L	Length
W	Width
MRI	Magnetic resonance imaging
SCO	Sertoli cells only tubules
